# Gallbladder metastasis of renal cell carcinoma presenting as a hypervascular polypoid lesion: case report of two cases with immunohistochemical analysis

**DOI:** 10.1186/s40792-020-00814-z

**Published:** 2020-04-28

**Authors:** Takuya Oba, Norihiro Sato, Toshihisa Tamura, Katsushi Fujimoto, Atsuji Matsuyama, Keiji Hirata

**Affiliations:** 1 Department of Surgery1, School of Medicine, University of Occupational and Environmental Health, Kitakyushu, 807-8555 Japan; 2 Department of Pathology and Oncology, School of Medicine, University of Occupational and Environmental Health, Kitakyushu, Japan

**Keywords:** Gallbladder metastasis, Renal cell carcinoma, Polypoid lesion of the gallbladder

## Abstract

**Background:**

Metastasis of renal cell carcinoma (RCC) to the gallbladder is rare, and its clinicopathological feature remains poorly understood. We here present two cases of gallbladder metastasis from RCC presenting as a hypervascular polypoid lesion.

**Case presentation:**

The first case was a 73-year-old man who had undergone right nephrectomy for clear cell RCC. Imaging studies detected a hypervascular polypoid lesion in the gallbladder 6 years after nephrectomy. Laparoscopic cholecystectomy was done. The pathological findings of the polypoid lesion showed proliferation of clear cells in the submucosal layer. Immunohistochemically, the tumor was positive for carbonic anhydrase 9 (CA9) but negative for cytokeratin 7 (CK7), suggestive of metastatic RCC. The second case was a 43-year-old man who had undergone right nephrectomy for clear cell RCC. Imaging studies revealed a hypervascular polypoid lesion of 20 mm in diameter in the gallbladder 1 year after nephrectomy. The patient underwent expanded cholecystectomy and extra-hepatic bile duct resection with lymphadenectomy. Microscopically, the polypoid lesion of the gallbladder was composed of clear cells in the submucosal layer. Immunohistochemical analysis showed positive staining for epithelial membrane antigen (EMA) and carcinoembryonic antigen (CEA) but negative staining for CK7, leading to the diagnosis of metastatic RCC.

**Conclusions:**

Gallbladder metastasis from RCC is rare but should be considered when a hypervascular polypoid lesion in the gallbladder is detected during the follow-up period after RCC treatment.

## Introduction

Gallbladder polyp is a common clinical entity being increasingly detected by screening or follow-up ultrasonography. Pathological diagnosis of gallbladder polyps includes cholesterol polyp, adenomatous polyp, hyperplastic polyp, inflammatory polyp, and gallbladder cancer. However, metastatic cancer presenting as a gallbladder polyp is extremely rare.

Hematological metastasis from renal cell carcinoma (RCC) occurs in a variety of organs, including lung, bone, lymph nodes, liver, adrenal, and brain [1]. Metastasis of RCC to the gallbladder is rare and is reported to be found in only 0.58% of autopsy cases of RCC [2]. Because of its rarity, the clinicopathological feature of gallbladder metastasis from RCC remains poorly understood.

In the present study, we present two cases of metachronous gallbladder metastasis from RCC which showed characteristic clinicopathological and immunohistochemical features.

## Case presentation

### Case1

A 73-year-old man had undergone laparoscopic right nephrectomy for early-stage RCC (clear cell type, T1bN0M0, stage I). A follow-up CT scan at 6 years after nephrectomy first detected a 6.4-mm polypoid lesion in the body of the gallbladder. A repeated CT in the following year (at 7 years after surgery) found the polypoid lesion rapidly grown to more than double in size. He was asymptomatic, and laboratory data (including CA19-9) showed no remarkable change.

**Fig. 1:**
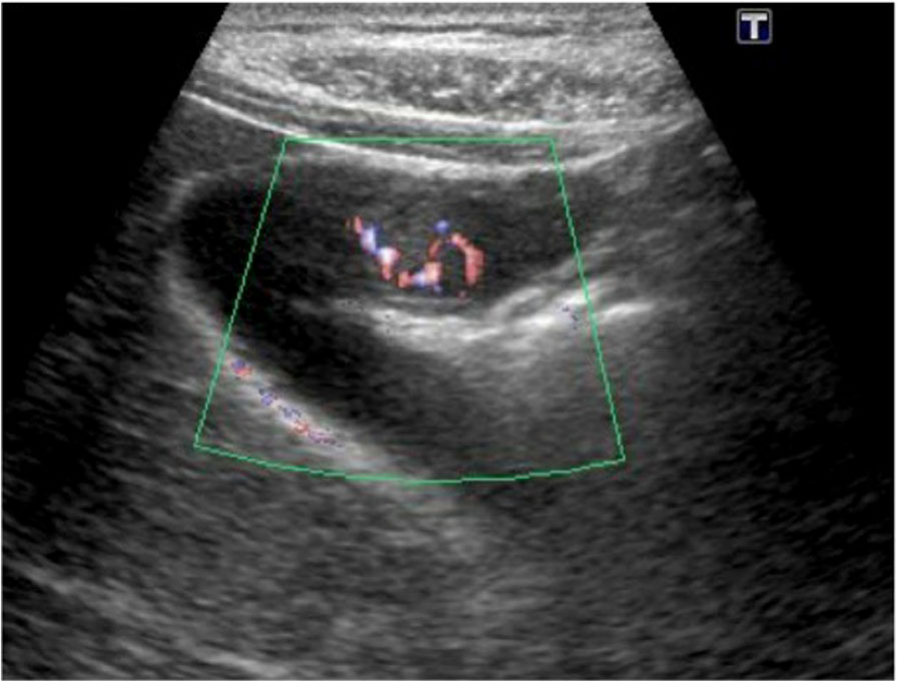
Color Doppler US images revealed hypervascular tumor of the gallbladder

On ultrasonography (US), the lesion was 13.9 × 10 mm and its surface was smooth. Color Doppler US images revealed hypervascularity of the lesion, suggesting its neoplastic nature (Fig. 1).

**Fig. 2:**
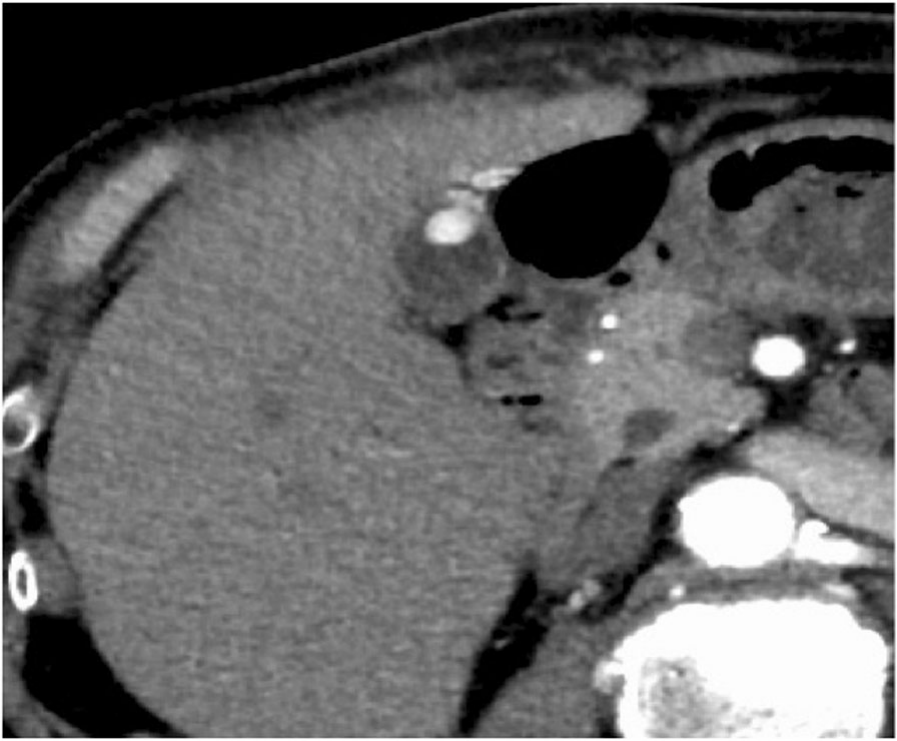
Dynamic CT scan showed a polypoid mass of the gallbladder. In arterial phase, it revealed very high and homogeneous contrast effect

A contrast-enhanced CT scan showed a polypoid mass in the gallbladder. In arterial phase, the lesion showed high and homogeneous contrast effect (Fig. 2).

Based on these clinical backgrounds and imaging findings, the possibility of gallbladder malignancy, including metastatic cancer from RCC, was considered.

We performed laparoscopic cholecystectomy. Before closing the skin incision, we placed an absorbable adhesion barrier (Interceed®) in case of being primary gallbladder carcinoma infiltrating the muscularis propria or beyond pathologically.

**Fig. 3:**
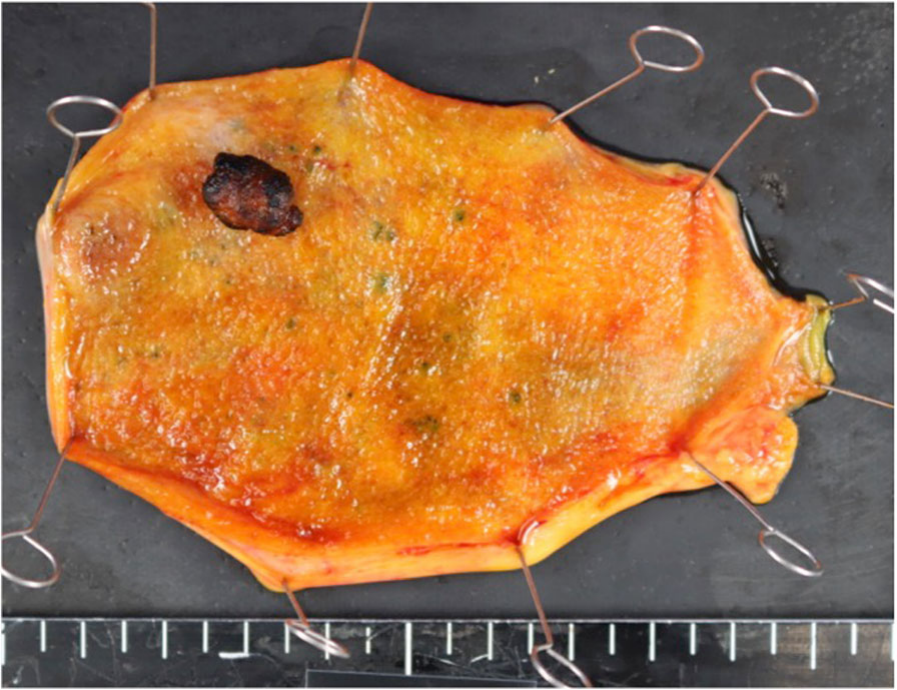
The specimen showed a pedunculated tumor in the body of the gallbladder

The specimen showed a pedunculated tumor in the body of the gallbladder with a dark brown appearance, suggesting hemorrhagic necrosis (Fig. 3).

**Fig. 4:**
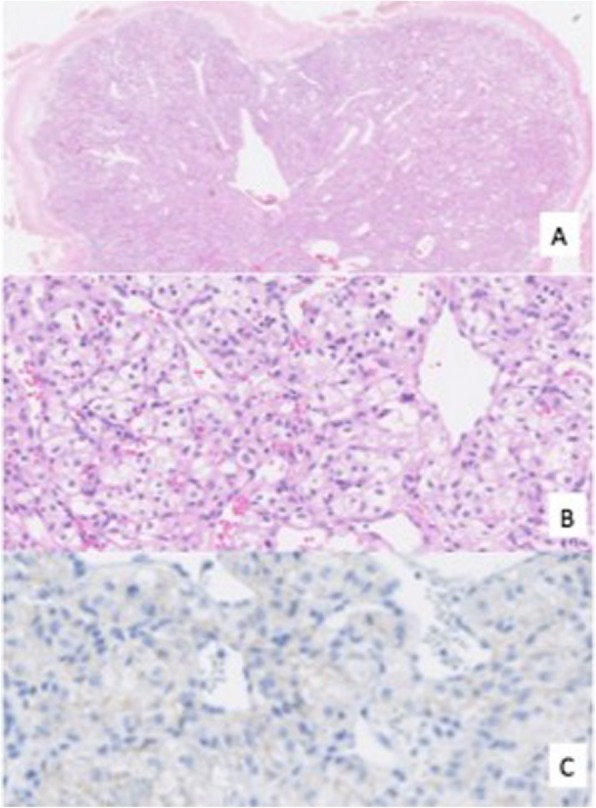
The tumor was composed of dense proliferation of neoplastic cells having clear cytoplasm in the submucosal layer covered by normal mucosal layer (**a** H&E × 12.5, **b** H&E × 200). Immunohistochemical analysis showed positive staining for CA9 in a membranous pattern (**c**)

Microscopically, the tumor was composed of proliferation of neoplastic cells having clear cytoplasm in an alveolar growth fashion in the submucosal layer covered by normal mucosal layer (Fig. 4a, b).

Immunohistochemical analysis showed positive staining for carbonic anhydrase 9 (CA9) but negative staining for cytokeratin 7 (CK7), consistent with clear cell carcinoma originating from the kidney (Fig. 4c). These pathological and immunohistochemical findings led to the diagnosis of gallbladder metastasis from RCC.

The patient is free from recurrence at 6 months after cholecystectomy.

### Case2

A 43-year-old man had undergone right nephrectomy for RCC (clear cell type, T2N0M1 (lung), stage IV). After nephrectomy, he was treated with interferon for lung metastasis. An abdominal CT scan at 1 year after nephrectomy showed a newly identified gallbladder lesion. He was asymptomatic, and laboratory data showed only slightly increased transaminase levels. Serum CEA and CA19-9 levels were within normal limits.

**Fig. 5:**
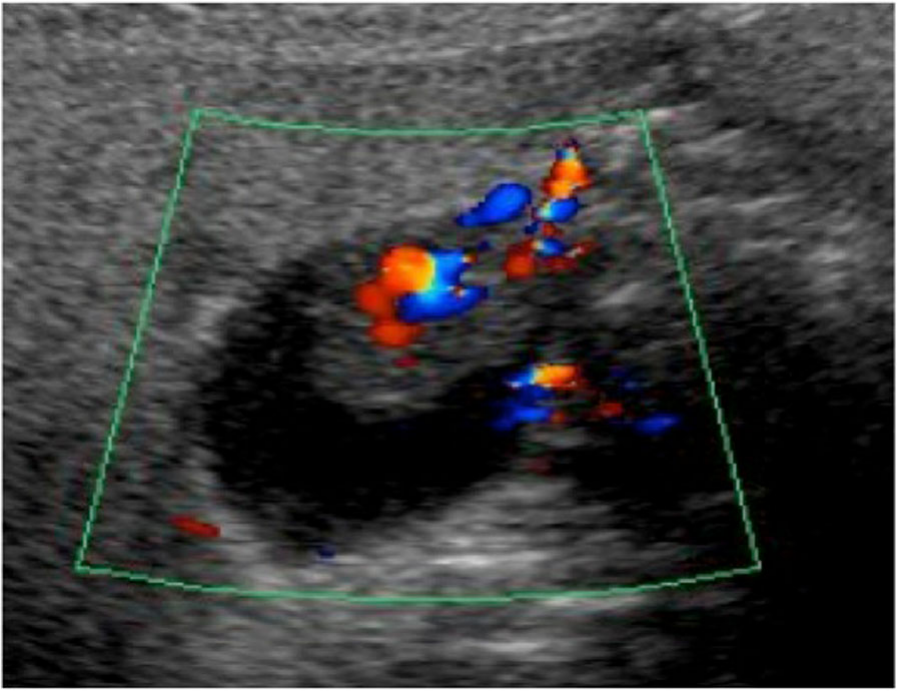
The lesion of the gallbladder was hypervascular by color Doppler US images

An ultrasonography showed a 14 × 26-mm polypoid lesion in the fundus of the gallbladder. The lesion was hypervascular by color Doppler US images (Fig. 5).

**Fig. 6:**
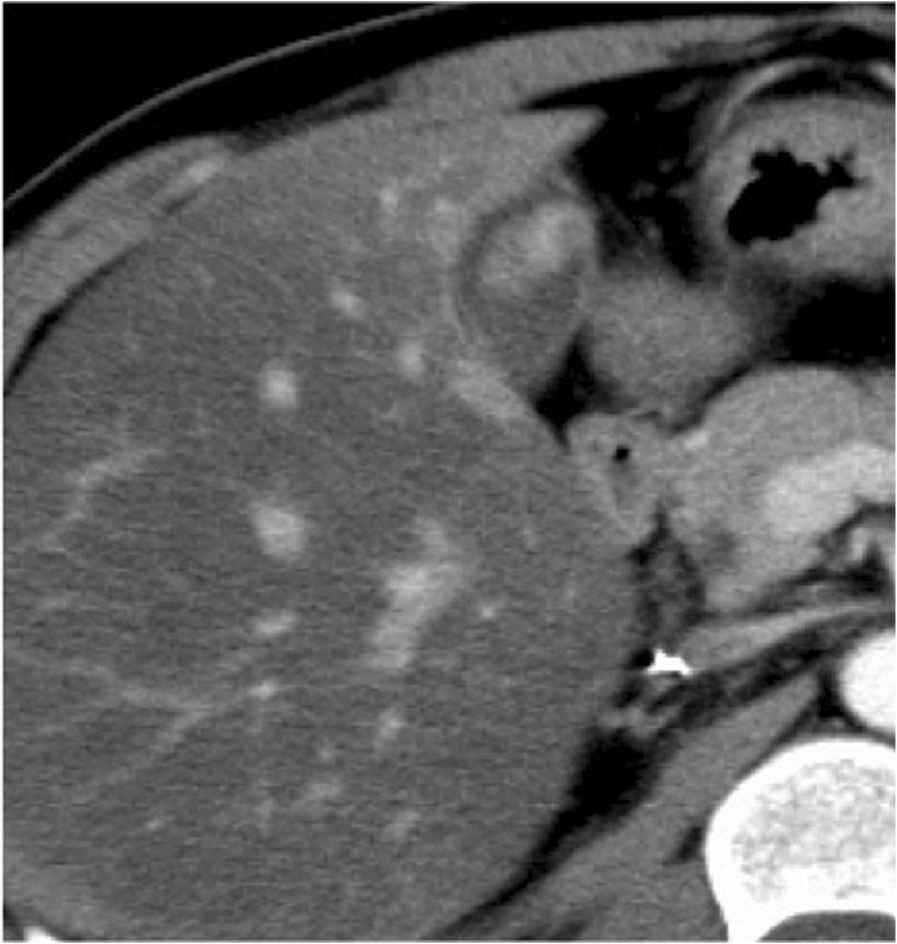
A contrast-enhanced CT scan showed a 20-mm lesion of the gallbladder with a contrast-enhanced effect

A contrast-enhanced CT scan showed a 20-mm lesion with a contrast enhanced effect (Fig. 6).

FDG-positron emission tomography (PET) showed intense accumulation of FDG in the gallbladder lesion.

On a tentative diagnosis of gallbladder cancer, we performed expanded cholecystectomy and extra-hepatic bile duct resection with lymphadenectomy.

**Fig. 7:**
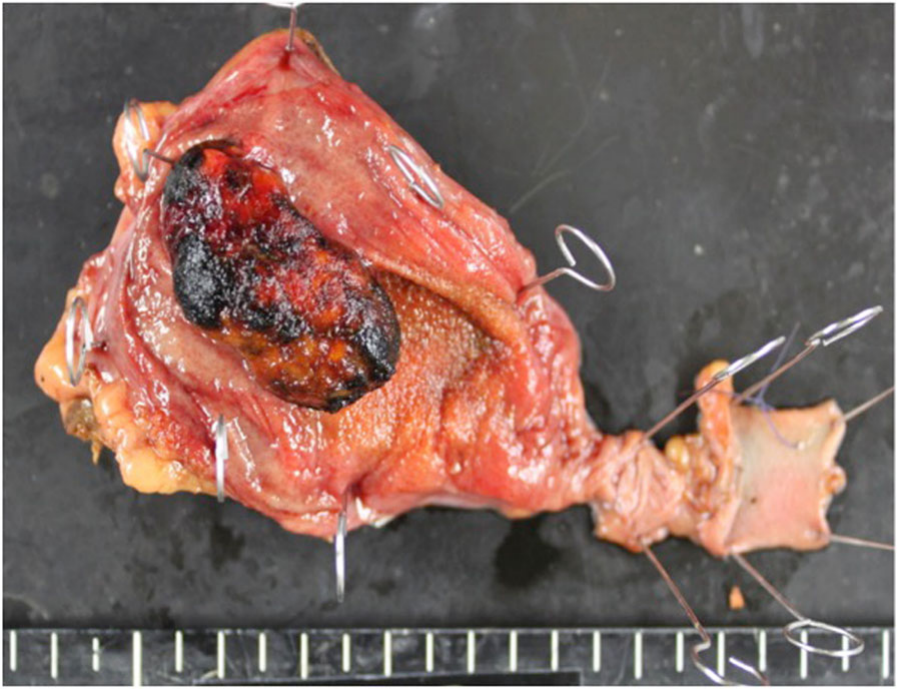
The specimen showed a 15 × 30-mm pedunculated tumor in the fundus of the gallbladder, with a dark brown appearance

The specimen showed a 15 × 30-mm pedunculated tumor in the fundus of the gallbladder, with a dark brown appearance (Fig. 7).

**Fig. 8:**
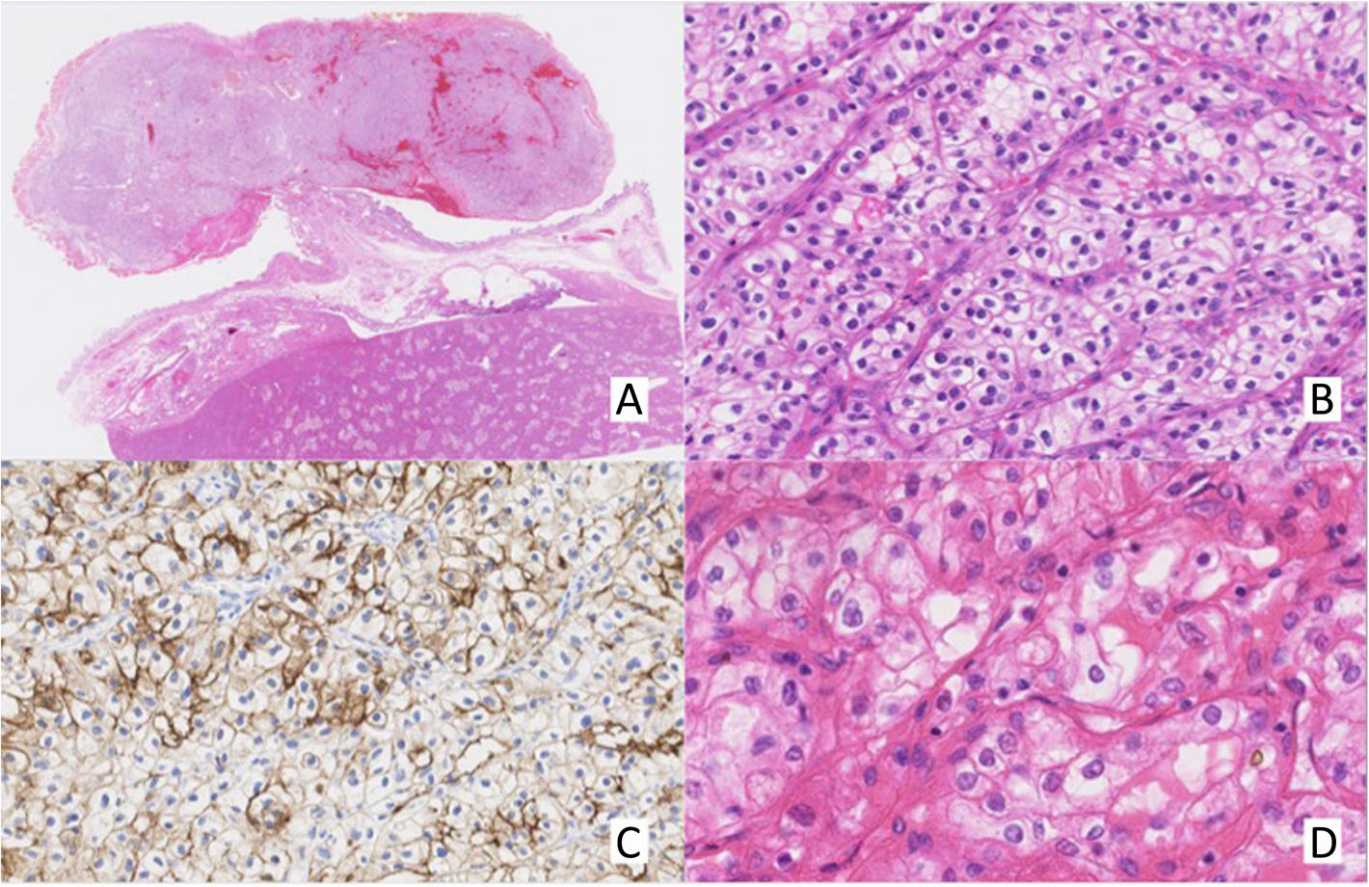
The tumor was composed of a hypercellular area in the submucosal layer. Tumor cells were homogenous and characterized by bright clear cytoplasm (**a** H&E × 4, **b** H&E × 250). Immunohistochemically, the tumor cells were positive for EMA (**c**). Histology of the primary lesion showed that cells with clear cytoplasm are growing in alveolar form (**d** H&E × 300)

Microscopically, the tumor was composed of a hypercellular area in the submucosal layer (Fig. 8a). Tumor cells were homogenous and characterized by bright clear cytoplasm (Fig. 8b). Adjacent mucosa had no dysplasia or carcinoma in situ. Immunohistochemically, the tumor cells were positive for epithelial membrane antigen (EMA) but negative for CK7 and carcinoembryonic antigen (CEA) (Fig. 8c). These immunohistochemical features were consistent with RCC. Based on these pathological and immunohistochemical findings, the gallbladder tumor was diagnosed as gallbladder metastasis from RCC.

He is alive at 7 years after extended cholecystectomy but found to have multiple metastatic tumors in the brain, bones, and pancreas.

## Discussion

In the present study, we presented two cases of gallbladder metastasis of RCC. These two cases have similar and peculiar clinicopathological features. First, imaging studies revealed a hypervascular polypoid lesion in the gallbladder. Second, gross appearance of the tumor was a pedunculated polyp with a thin stalk and a dark brown surface. Third, the tumor was composed of massive proliferation of clear cells in the submucosal layer. These findings may provide a clue to the diagnosis of gallbladder metastasis from RCC.

Because of its rarity, the clinicopathological feature of gallbladder metastasis of RCC remains poorly understood. In an attempt to elucidate its clinicopathological feature, we reviewed previously reported cases. A search for Japanese literature using Ichushi-Web and PubMed (1990–2019, keywords: Renal cell carcinoma, Gallbladder metastasis) revealed 46 reported cases of gallbladder metastasis from RCC including our present cases (Table 1). There were 37 male and 9 female patients with an average age of 64.8 years. In most cases, patients were asymptomatic, and the gallbladder lesion was incidentally detected by follow-up or screening US or CT. The median maximum diameter of the lesion was 18 mm (range, 5–75 mm). These included synchronous metastasis in 12 patients and metachronous metastasis in 34 patients. The median duration to detect gallbladder metastasis from nephrectomy was 6 years (range, 0.2–27 years) in metachronous cases. Interestingly, metastasis of renal cell carcinoma to the gallbladder can occur even at an early stage of primary tumor. Among the 19 cases of metachronous metastasis whose primary stage was reported in the literature, 11 patients were stage I. The most frequent macroscopic demonstration was pedunculated lesion in 37 patients (20%). Twenty-eight patients (61%) showed metastasis to other organs including the lung (16 patients), bones (6 patients), brain (4 patients), pancreas (4 patients), and contralateral kidney (4 patients). During a median follow-up period of 10 months, 31 patients (67%) were alive and 6 patients (13%) died from metastatic disease. The presence or absence of metastasis to other organs might determine the survival time in these patients [3, 4].

**Table 1:** A search for Japanese literature showed 46 reported cases of gallbladder metastasis from RCC

Parameter	No. of patients (%)	Median (range)
Sex	Male, 37 (81)	
	Female, 9 (19)	
Age		64.8 years (43–80)
Symptom	+, 10 (22)	
	−, 36 (78)	
Timing	Syn, 12 (26)	
	Meta, 34 (74)	6 years (0.2–27)^a^
Stage^b^	I, 11 (23.9)	
	II, 5 (10.9)	
	III, 3 (6.5)	
	IV, 13 (28.3)	
	Not reported, 14 (30.4)	
Operation procedure	EC, 8 (17.4)	
	OC, 25 (54.3)	
	LC, 13 (28.3)	
Maximum diameter		18 mm (5–75)
Macroscopic findings	Peduncuated, 37 (80)	
	Mass, 9 (20)	
Metastasis to other organs	+, 28 (61)	
	−, 18 (39)	
Outcome	Alive, 31 (67)	10 months (1–96)^c^
	Dead, 6 (13)	
	Not reported, 9 (20)	

*Syn* synchronous, *Meta* metachronous, *EC* extended cholecystectomy, *OC* open cholecystectomy, *LC* laparoscopic cholecystectomy

^a^Duration to detect metachronous gallbladder metastasis from nephrectomy

^b^Post-nephrectomy staging

^c^Follow-up period

On ultrasonography, the tumor often has a hyperechoic band on its surface because of its submucosal nature [4], though our present cases did not show such a clear hyperechoic band. In our present cases, color Doppler US and contrast-enhanced CT showed hypervascularity within the tumor, which may reflect the histological nature of RCC. However, it is difficult to distinguish between primary gallbladder neoplasm (including adenocarcinoma and neuroendocrine tumor) and metastatic tumor based on these imaging findings alone.

There is no definitive treatment strategy for gallbladder metastasis of RCC. Clinical Practice Guideline for Renal Cell Carcinoma in Japan reviewed that metastasectomy is expected to improve survival for RCC patients if they have good performance status and long disease-free period, and complete resection is possible [5]. In this context, simple cholecystectomy may be enough to completely remove the gallbladder metastasis located within the submucosal layer [6, 7]. However, selection of optimal surgical procedure is sometimes difficult because the primary gallbladder cancer cannot be ruled out preoperatively.

Microscopically, primary clear cell carcinoma of the gallbladder tends to have a component of conventional adenocarcinoma and have dysplasia or carcinoma in situ in adjacent mucosa with H&E stain [8].

In the present study, we used immunohistochemical analysis to distinguish between primary gallbladder cancer and metastasis of RCC. In general, primary gallbladder carcinoma consistently shows positive staining for CK7, EMA, and CEA, but is negative for CA9 and vimentin [9–11]. On the contrary, metastatic clear cell RCC shows the opposite staining pattern [12]. These immunohistochemical findings might help in the differential diagnosis.

## Conclusions

We present two cases of gallbladder metastasis of RCC. Gallbladder metastasis of RCC should be considered when a hypervascular polypoid lesion in the gallbladder is detected during systemic screening of RCC or the follow-up period after RCC treatment.

## Data Availability

The data are not available for public access because of patient privacy concerns but are available from the corresponding author on reasonable request.

## Abbreviations

RCC: Renal cell carcinoma
CA9: Carbonic anhydrase 9
CK7: Cytokeratin 7
EMA: Epithelial membrane antigen
CEA: Carcinoembryonic antigen
CT: Computed tomography
US: Ultrasonography
CA19-9: Carbohydrate antigen 19-9
FDG-PET: Fluorodeoxyglucose-positron emission tomography

## References

[1] Jun G, Manuel CM (2016). Metastasis in renal cell carcinoma: biology and implications for therapy. Asian J Urol.

[2] Weiss L, Harlos JP (1988). Metastatic patterns of renal carcinoma: an analysis of 687 necropsies. J Cancer Res Clin Oncol.

[3] Chung PH (2012). Renal cell carcinoma with metastasis to the gallbladder: four cases from the National Cancer Institute (NCI) and review of the literature. Urol Oncol.

[4] Takagi K, Kawase K (2019). Gallbladder metastasis from renal cell carcinoma: a case report and literature review. Urol Case Rep.

[5] Naito S, Kinoshita H, et al. Prognostic factors of patients with metastatic renal cell carcinoma with removed metastases: a multicenter study of 556 patients. Urology. 2013;82:846–51. 10.1016/j.urology.2013.06.035 24074981

[6] Saito Y (2018). Gallbladder metastasis of renal clear cell carcinoma 15 years after primary cancer excision: a case report. J Med Case Rep.

[7] Kavolius JP, Mastorakos DP (1998). Resection of metastatic renal cell carcinoma. J Clin Oncol.

[8] Deepali J, Prem C (2013). Metastasis renal cell carcinoma of gall bladder. Saudi J Kidney Dis Transpl.

[9] Mitsimponas N, Crespo F (2017). Isolated gallbladder metastasis from renal cell carcinoma : a case report and review of the literature. Ann Hematol Oncol.

[10] Li G (2017). CA9 as a biomarker in preoperative biopsy of small solid renal masses for diagnosis of clear cell renal cell carcinoma. Biomarkers..

[11] Jiang Y, Su G (2015). Clinicopathological features and prognosis of renal cell carcinoma with sarcomatoid differentiation. Zhonghua Zhong Liu Za Zhi.

[12] Brittinger A, Alterkrugar I, Barth P (1995). Clear cell carcinoma of gallbladder, a histological and immunestological study. Pathol Res Pract.

